# Doxycycline induces bone repair and changes in Wnt signalling

**DOI:** 10.1038/ijos.2017.28

**Published:** 2017-09-29

**Authors:** Kátia do Nascimento Gomes, Ana Paula Negreiros Nunes Alves, Paula Góes Pinheiro Dutra, Glauce Socorro de Barros Viana

**Affiliations:** 1 Faculty of Dentistry, Pharmacy and Nursing, Federal University of Ceará, Fortaleza, Brazil; 2 Faculty of Medicine, Federal University of Ceará, Fortaleza, Brazil

**Keywords:** alveolar bone loss, bone diseases, bone repair, doxycycline, Dickkopf-1, immunohistochemistry, Wnt-10b

## Abstract

Doxycycline (DOX) exhibits anti-inflammatory and MMP inhibitory properties. The objectives of this study were to evaluate the effects of DOX on alveolar bone repair. Controls (CTL) and DOX-treated (10 and 25 mg·kg^−1^) molars were extracted, and rats were killed 7 or 14 days later. The maxillae were processed and subjected to histological and immunohistochemical assays. Hematoxylin-eosin staining (7th day) revealed inflammation in the CTL group that was partly reversed after DOX treatment. On the 14th day, the CTL group exhibited bone neoformation, conjunctive tissue, re-epithelization and the absence of inflammatory infiltrate. DOX-treated groups exhibited complete re-epithelization, tissue remodelling and almost no inflammation. Picrosirius red staining in the DOX10 group (7th and 14th days) revealed an increased percentage of type I and III collagen fibres compared with the CTL and DOX25 groups. The DOX10 and DOX25 groups exhibited increases in osteoblasts on the 7th and 14th days. However, there were fewer osteoclasts in the DOX10 and DOX25 groups on the 7th and 14th days. Wnt-10b-immunopositive cells increased by 130% and 150% on the 7th and 14th days, respectively, in DOX-treated groups compared with the CTL group. On the 7th day, Dickkopf (Dkk)-1 immunostaining was decreased by 63% and 46% in the DOX10 and DOX25 groups, respectively. On the 14th day, 69% and 42% decreases in immunopositive cells were observed in the DOX10 and DOX25 groups, respectively, compared with the CTL group. By increasing osteoblasts, decreasing osteoclasts, activating Wnt 10b and neutralising Dkk, DOX is a potential candidate for bone repair in periodontal diseases.

## Introduction

Bone is a complex mineralised connective tissue characterised by constant remodelling, involving cycles of bone resorption and bone formation. Bone tissue has considerable potential for healing, exhibiting cooperative action among bone forming and resorptive cells to restore the architecture and function of damaged tissue.^1, 2, 3^ The bone healing process is triggered by injury, which results in a local inflammatory immune reaction. The development of this reaction highly influences the outcome of the healing process. Among the specialized bone tissues, the alveolar bone, which supports the tooth in the maxilla and mandible, is characterised by distinctive features, such as continuous and rapid remodelling in response to stimuli by force.^4, 5^

Recent evidence^6^ has indicated the histomorphometric and molecular profiles of alveolar bone healing after tooth extraction. That study has shown that after the initial clot dominance, immature granulation tissue development is evident by the 7th day. This development is characterised by marked cell proliferation, angiogenesis and inflammatory cell infiltration associated with growth factors, cytokines, chemokines and receptors and the expression of mesenchymal stem cell markers. On the 14th day after tooth extraction, the immature granulation tissue is replaced by mature connective tissue, which is characterised by decreased inflammatory infiltrate, increased bone formation, the expression of matrix remodelling enzymes (matrix metalloproteinase (MMP)-2 and MMP-9), markers of bone formation/maturation, and chemokines and receptors associated with healing.

In addition to inhibiting MMP activity,^7, 8^ doxycycline (DOX) potentially decreases enzyme expression at the transcriptional level. Furthermore, DOX activity toward mammalian collagenases and gelatinases appears to be unrelated to its antimicrobial efficacy. Others have shown that DOX inhibits not only MMP-8 and MMP-9 (gelatinase B) activity, but also the synthesis of MMPs in human endothelial cells.^9^ A later study has concluded that the inhibition of MMPs by tetracyclines, including minocycline, a second generation tetracycline similar to DOX, occurs mainly via the down regulation of respective gene expression.^10^

MMPs degrade virtually all constituents of the extracellular matrix. MMPs are critical for connective tissue remodelling and healing as well as bone healing after tooth extraction.^11, 12^ The molecular environment of chronic wounds contains high levels of pro-inflammatory cytokines and MMPs, which impair normal healing. This process is positively affected by DOX.^13, 14^ Previously, the systemic administration of DOX has been found to prevent both root resorption and bone loss in rats.^15^

Furthermore, the Wnt signalling pathway (involving proteins that pass signals into a cell through cell surface receptors) plays a key role in bone development and homeostasis. Wnt ligands promote bone growth, thereby leading to the hypothesis that Wnt signalling activation may stimulate bone healing.^16^ Dysregulation of this pathway greatly inhibits bone formation and the healing process, thus suggesting that this pathway has an essential role in bone regeneration.^17^ Wnt signalling is activated by wounding and participates in every stage of the healing process, from the control of inflammation and programmed cell death to the mobilisation of stem cell reservoirs within the wound site.^18^

Furthermore, signalling via the classical Wnt pathway is critical for bone deposition and bone remodelling. Among other mechanisms, this signalling pathway is regulated by Dickkopf proteins (Dkks), which bind and promote the internalisation of lipoprotein receptor-related protein (LRP)5 or LRP6. Blocking these Wnt receptor components effectively downregulates Wnt signalling. Dkk has been implicated in bone formation and bone diseases. The induction of the Wnt signalling pathway promotes bone formation, whereas its inactivation by Dkk leads to osteopenic states.^19^

Given the anti-inflammatory and MMPs inhibitory properties of DOX, the objectives of the present work were to use histological and histomorphometric methods to evaluate the effects of DOX on alveolar bone repair after tooth extraction in rats. In addition, we performed immunohistochemistry detecting Wnt-10b and its inhibitor Dkk-1.

## Materials and methods

### Drugs

Ketamine hydrochloride was purchased from Holliday-Scott SA, Argentina, and xylazine was obtained from Kensol Konig, Brazil. Doxycycline hydrochloride was obtained from EMS Laboratory, São Paulo, Brazil. Antibodies to Wnt-10b and Dkk-1 were obtained from Santa Cruz Biotechnology Inc., Dallas, Texas, or from ABCAM Inc., Cambridge, MA, USA. All other drugs and reagents were of analytical grade.

### Animals and experimental design

Male Wistar rats (150–200 g) from the Animal House of the Federal University of Ceará (UFC) were maintained in appropriated cages (five animals per cage) and given free access to water and food. The study protocol was submitted to and approved by the UFC Institutional Ethics Committee and was performed according to the Guide for the Care and Use of Laboratory Animals (National Institutes of Health (NIH), USA, 2011).

### Tooth extractions

The animals (*n*=5 per group) were divided into the following groups:two control (CTL) groups treated with saline for 7 or 14 days and four groups of DOX-treated rats, two of which were treated with DOX at doses of 10 or 25 mg·kg^−1^, p.o., once daily for 7 days, and two of which were treated with the same doses of DOX for 14 days. The treatments started 1 h before the surgical procedure and continued for 7 or 14 days. For tooth extractions, the animals were anaesthetized with ketamine (80 mg·kg^−1^, i.p.) and xylazine (10 mg·kg^−1^, i.p.). Each animal was placed in the dorsal decubitus position, and the mouth was kept open with rubber bands. The upper molars on the right side were extracted using a lever movement with a 3S Hollemback spatula adapted to the size of the teeth. During the surgical procedure, the area was irrigated continuously with saline. After the extraction, the surgical site was subjected to gauze compression to avoid haemorrhage and to aid in clot removal. After 7 and 14 post-surgery days, the animals were euthanized via decapitation. The maxillas were removed and fixed in buffered formol for 48 h. Then, hemi-maxillas were separated and demineralised with a 10% buffered EDTA solution for 30 days and were processed for histological (hematoxylin-eosin (HE) staining) and immunohistochemical assays for Wnt-10b and Dickkopf (Dkk)-1.

### Hematoxylin/eosin histological analyses

From each animal, different alveolar regions (coronal, middle and apical) and slices from the left hemi-maxilla (no tooth extraction),which corresponded to the same region of the first left superior molar, were obtained. For qualitative analyses, the following parameters were analysed: polymorphonuclear and mononuclear cell inflammatory infiltrates, conjunctive tissue remodelling and number of osteoclasts. For these analyses, the Particle Analysis-Cell Counter and ImageJ software (NIH, USA) were used. The analyses were performed at the 7th and 14th days after the surgical procedure.

### Histological analyses for picrosirius red

Picrosirius red (PSR) staining was used to evaluate the types of collagen fibres that were present in the tissue and involved in alveolar bone repair. Under appropriate conditions, type I collagen fibres appear with a reddish-yellow colour, whereas type III collagen fibres are greenish. In the present work, the analyses were performed at the 7th and 14th days after the tooth extraction using both non-polarised and polarised images, and the data quantification was performed with the ImageJ software (NIH, USA).

### Immunohistochemistry assays

Hemi-maxilla slices (5 μm) from animals of all study groups were fixed in 10% buffered formol, for 48 h, followed by 70% alcohol. The sections were embedded in paraffin wax, and slices were processed on appropriate glass slides. These samples were placed in an oven at 58 °C for 10 min, then subjected to deparaffinization in xylol, rehydration in alcohol at decreasing concentrations and washing in distilled water and phosphate buffered saline (PBS) (0.1 mol·L^−1^ sodium phosphate buffer, pH 7.2) for 10 min. Endogenous peroxidase was blocked with a 3% hydrogen peroxide solution (20 min), and protein was blocked with 5% BSA for 40 min. The slices were incubated overnight with the primary anti-goat Wnt-10b antibody (1:100 dilution) or a polyclonal anti-rabbit Dkk-1 antibody (1:200 dilution). After being washed with PBS, the slices were incubated with the biotinylated anti-rabbit secondary antibody (1:400 dilution) for 30 min. After being washed, the slices were incubated with the streptavidin–peroxidase-complex for 30 min, washed again with PBS and stained with 0.1% DAB (in 3% hydrogen peroxide). Finally, the glass slides were washed in distilled water and counterstained with Mayer’s hematoxylin, washed in tap water, dehydrated in alcohol (at increasing concentrations), diaphonized in xylol and mounted on Entelan for optic microscopy examination. The immunostaining intensity was quantified with the ImageJ software (NIH, USA).

### Statistical analyses

For parameters analysed in HE staining, scores were calculated, and the data were subjected to the non-parametric Kruskal–Wallis test, followed by the Dunn *post hoc* test. The results are presented as median and extreme values. The results (means±s.e.m.) of the histomorphometric analyses and quantitative variables were subjected to two-way ANOVA, followed by the Tukey *post hoc* test. The results were considered significant at *P*<0.05.

## Results

### Histological descriptive analyses

#### HE analyses

**Figure 1:**
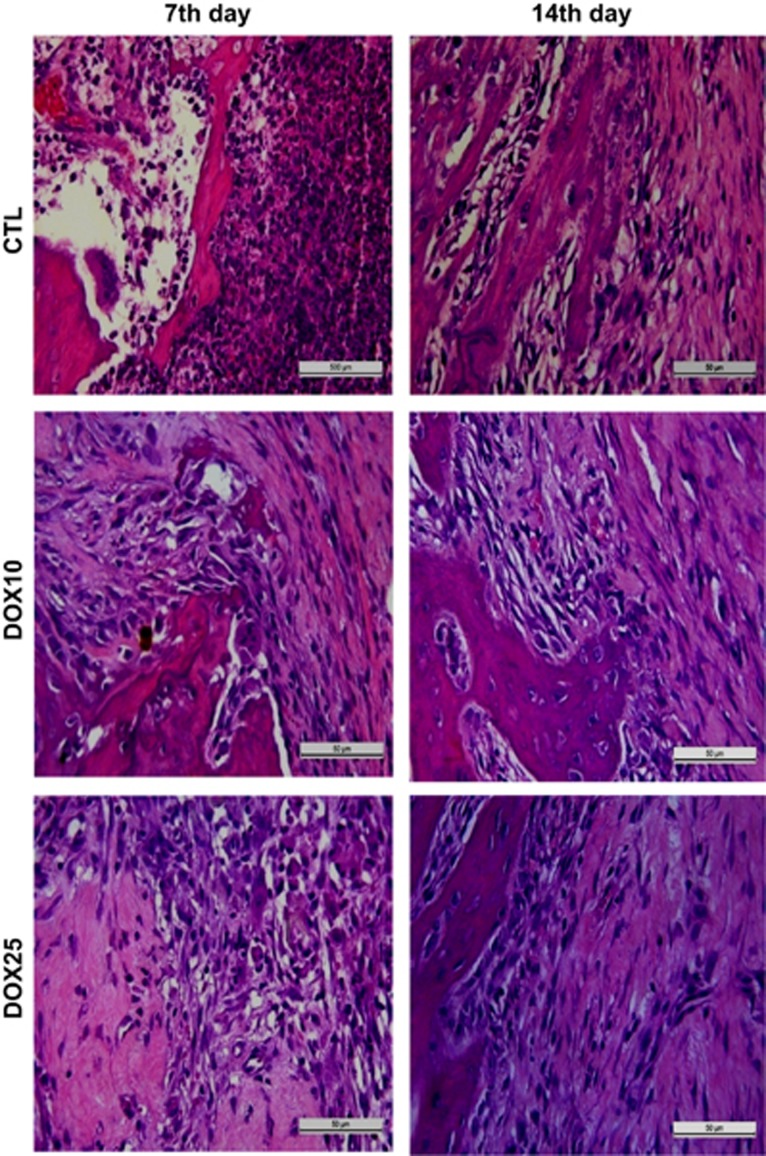
**Representative photomicrographs of alveolar tissues after tooth extraction.** On the 7th day, the CTL group exhibited no healing and significant inflammatory infiltrate and fibroblastic proliferation. The DOX10 and DOX25 groups exhibited the absence of inflammatory PMN cells, but mononuclear inflammatory infiltrates and remodelling signals were present. On the 14th day, the CTL group exhibited bone neoformation, re-epithelization and the absence of inflammatory infiltrate. After DOX treatment, complete re-epithelization and remodelling were observed, but an inflammatory infiltrate and numerous osteoblasts were present. HE staining, × 400. CTL, control; DOX10, doxycycline 10 mg·kg^−1^; DOX25, doxycycline 25 mg·kg^−1^.

On the 7th day after surgery, the CTL group (Figure 1) exhibited alveoli with no healing that were completely filled with granulation tissue and blood vessels. The conjunctive tissue presented intense inflammatory infiltrates with a predominance of polymorphonuclear (PMN) cells, mainly neutrophils, and fibroblastic proliferation. The DOX10 group exhibited alveoli with the absence of discrete PMN cell infiltrate. The conjunctive tissue presented initial signals of remodelling with osteoid tissue. However, this group exhibited an intense mononuclear inflammatory infiltrate and numerous osteoblasts and osteoclasts. The DOX25 group exhibited a pattern similar to that of the DOX10 group, except for a decrease in the mononuclear inflammatory infiltrate, blood vessels, and osteoclasts, as well as discrete conjunctive tissue remodelling. On the 14th day after surgery the CTL group exhibited bone neoformation, dense fibrous conjunctive tissue, partial re-epithelization and the absence of inflammatory infiltrate. The DOX10 group exhibited complete re-epithelization, significant conjunctive tissue remodelling, and the formation of osteoid tissue. This group also contained osteoblasts and almost no inflammatory infiltrate. The DOX25 group exhibited complete re-epithelization, significant conjunctive tissue remodelling and more trabecular bone than osteoid tissue. In addition, the DOX25 group contained numerous osteoblasts and minimal inflammatory infiltrate and blood vessels.

#### Picrosirius red staining

**Figure 2:**
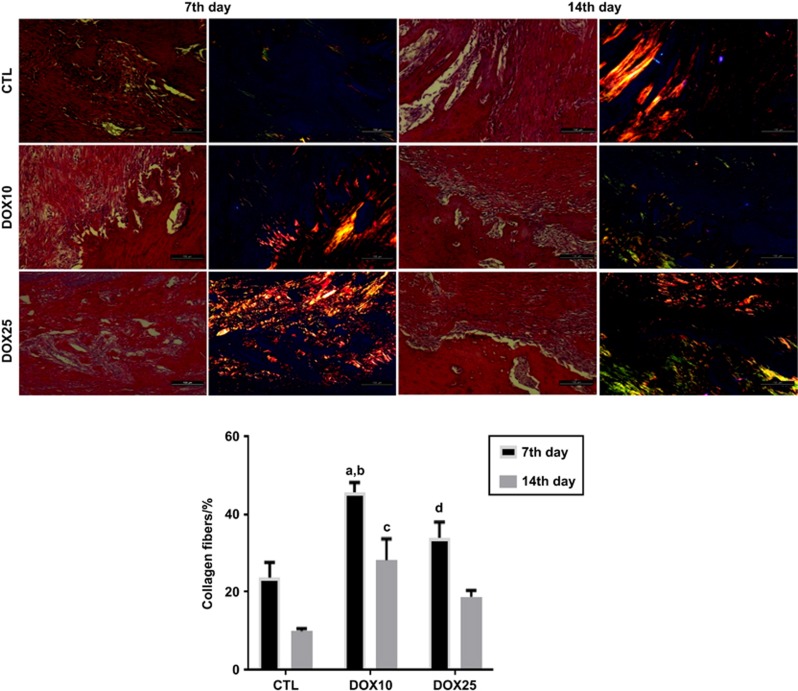
**Representative photomicrographs of alveolar tissues after picrosirius red (PSR) staining (× 200), on the 7th and 14th post-tooth extraction days.** The non-polarised (1st and 3rd columns) and polarised (2nd and 4th columns) light images are presented in the upper panel. The percentages of collagen fibres (lower panel) were determined from the polarised light images. (**a**) *vs* CTL, 7th day, *P*=0.000 5; (**b**) *vs* DOX10, 14th day, *P*=0.008 6; (**c**) *vs* CTL, 14th day, *P*=0.005 4; (**d**) *vs* DOX25, 14th day, *P*=0.031 8 (Two-way ANOVA and Tukey test for multiple comparisons). CTL, control; DOX10, doxycycline 10 mg·kg^−1^; DOX25, doxycycline 25 mg·kg^−1^.

The analyses on the 7th post-surgery day revealed decreased collagen deposition in the three groups. However, in the CTL, the predominance of type III collagen fibres was noted, a result that differed from those in the DOX-treated groups, which exhibited a predominance of type I fibres. In addition, increases in collagen fibres were observed in the DOX10 and DOX25 groups (170% and 130%, respectively) compared with the CTL group. On the 14th day after surgery, the CTL group exhibited bone matrix deposition with minimal type III fibres. At that time point, the DOX25 groups respectively exhibited 290% and 190% increases in collagen fibres, as compared with the CTL group. Although type III predominated in the DOX10 group, a predominance of type I collagen fibres was observed in the DOX25 group compared with the DOX10 and CTL groups (Figure 2).

### Histomorphometric analyses

#### PMN inflammatory infiltrate

On the 7th post-surgery day (Table 1), the CTL group presented alveoli with intense inflammatory infiltrate (median 3: 3–3). This pattern was significantly reversed in the DOX10 (median 1: 0–1) and DOX25 groups (median 1: 0–2). On the 14th day after surgery, the intensity of the inflammatory infiltrate did not significantly change in the DOX10 (median 3: 2–3) and DOX25 (median 3: 3–3) groups compared with the CTL group (median 3: 3–3).

#### Mononuclear inflammatory infiltrate

On the 7th post-surgery day (Table 2), no differences were observed in this parameter among the CTL (median 3: 3–3), DOX10 (median 3: 1–3) or DOX25 (median 2: 1–2) groups. On the 14th after surgery day, all three groups exhibited a significant decrease in the intensity of mononuclear inflammatory infiltrate: CTL (median 3: 3–3), DOX10 (median1: 0–1) and DOX25 (median1: 0–1).

#### Alveolar conjunctive tissue remodelling

On the 7th day after tooth extraction, a significant degree of remodelling was observed in the groups treated with DOX10 (median 1:1–2) and DOX25 (median 1:1–1) compared with the CTL group (median 0:0–1). However, on the 14th day, the degree of remodelling in the group treated with 25 mg·kg^−1^ DOX increased (median 2: 2–3) compared with that in the DOX10 (median1:1–1) and CTL (median 1:1–1) groups (Table 3).

#### Qualitative and quantitative analyses of osteoblasts

**Figure 3:**
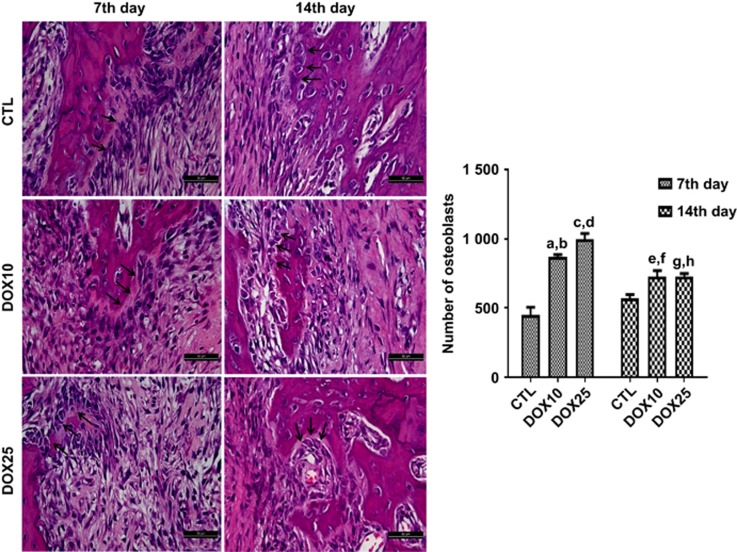
**Representative HE staining (× 400) photomicrographs of alveolar tissue on the 7th and 14th days after tooth extractions.** For both protocols, there was a significant increase in the number of osteoblasts (black arrows) after DOX treatment compared with that in the CTL. (**a**, **b**) *vs* CTL (7th and 14th days), *P*<0.000 1 and *P*=0.000 9, respectively; (**c**, **d**) *vs* CTL (7th and 14th days), *P*<0.000 1 and *P*<0.000 1, respectively; (**e**, **f**) *vs* CTL (7th day) and *vs* DOX25 (7th day), *P*<0.001 7 and *P*<0.002 3, respectively; (**g**, **h**) *vs* CTL (7th day) and *vs* DOX25 (7th day), *P*<0.001 9 and *P*<0.002 0, respectively (Two-way ANOVA and Tukey test for multiple comparisons). HE, hematoxylin-eosin; CTL, control; DOX10, doxycycline 10 mg·kg^−1^; DOX25, doxycycline 25 mg·kg^−1^.

Representative photomicrographs of alveolar tissues from the three study groups indicated the presence of osteoblasts on the 7th and 14th days (Figure 3) after tooth extraction. Data quantification revealed that 10 and 25 mg·kg^−1^ DOX significantly increased the number of osteoblasts by 190% and 220%, respectively, compared with that in the CTL group on the 7th day after-tooth-extraction. Similar increases (130%) were observed for both the DOX10 and DOX25 groups on the 14th day, as compared with CTL.

#### Qualitative and quantitative analyses of osteoclasts

**Figure 4:**
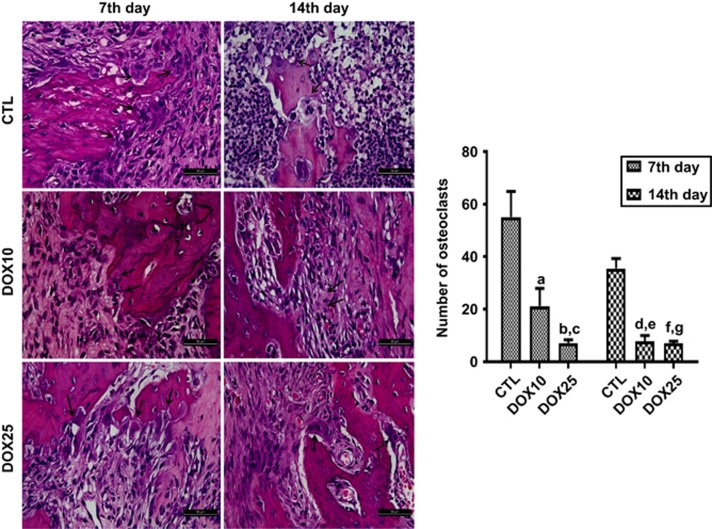
**Representative HE staining photomicrographs (× 400) of alveolar tissue on the 7th and 14th days after tooth extraction.** For both protocols, there was a significant decrease in the number of osteoclasts (black arrows) after DOX treatment, as compared with that in the CTL. (**a**) *vs* CTL (7th day), *P*=0.006 0; (**b**, **c**) *vs* CTL (7th and 14th days), *P*<0.000 3 and *P*<0.021 4, respectively; (**d**, **e**) *vs* CTL (7th and 14th days), *P*<0.000 4 and *P*<0.024 9, respectively; (**f**, **g**) *vs* CTL (7th and 14th days), *P*<0.000 3 and *P*<0.000 3, respectively (Two-way ANOVA and Tukey test for multiple comparisons). HE, hematoxylin-eosin; CTL, control; DOX10, doxycycline 10 mg·kg^−1^; DOX25, doxycycline 25 mg·kg^−1^.

Figure 4 shows representative photomicrographs of alveolar tissues from the three study groups, indicating the presence of osteoclasts on the 7th and 14th days after tooth extraction. The data quantification revealed 62% and 87% decreases in the number of osteoclasts in the groups treated with 10 and 25 mg·kg^−1^ DOX, respectively, compared with the CTL group on the 7th day. A similar pattern was observed on the 14th day, with decreases of 78% and 80% in the DOX10 and DOX25 groups, respectively, compared with CTL.

#### Percentage of bone tissue

**Figure 5:**
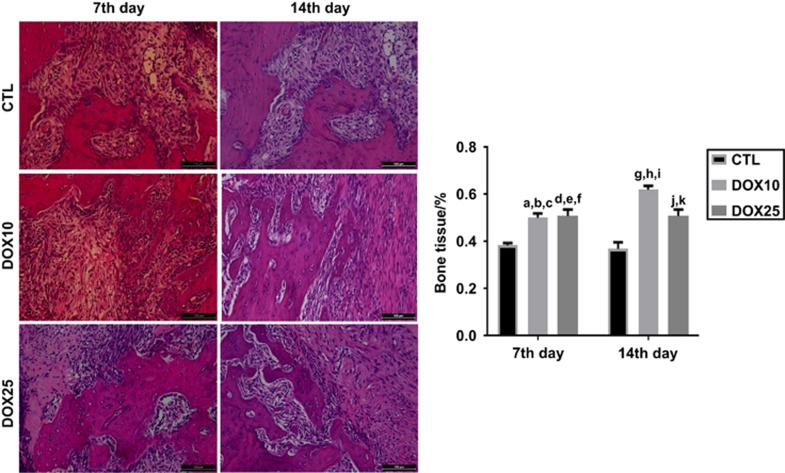
**Representative HE staining photomicrographs (× 400) revealing significant increases in the percentage of bone tissue after DOX treatment, as compared with that in the CTL, on the 7th and 14th days after tooth extraction.** (**a**, **b**) *vs* CTL (7th and 14th days) and (**c**) *vs* DOX10 (14th day), *P*=0.013 5, *P*=0.004 9 and *P*=0.011 6, respectively; (**d**, **e**) *vs* CTL (7th and 14th days) and (**f**) *vs* DOX10 (14th day), *P*=0.008 1, *P*=0.003 0, *P*=0.019 3, respectively; (**g**, **h**) *vs* CTL (7th and 14th days) and (**i**) *vs* DOX25 (14th day), *P*=0.000 1, *P*=0.000 1, *P*=0.019 3, respectively; (**j**, **k**) *vs* CTL (7th and 14th days), *P*=0.008 1, *P*=0.003 0, respectively (Two-way ANOVA and Tukey test for multiple comparisons). HE, hematoxylin-eosin; CTL, control; DOX10, doxycycline 10 mg·kg^−1^; DOX25, doxycycline 25 mg·kg^−1^.

Similar and significant increases (~130%) were observed for this parameter in the groups treated with DOX at both doses, on the 7th day (compared with the CTL group). On the 14th day (Figure 5), increases of 170% and 140% were observed in the DOX10 and DOX25 groups, respectively.

### Immunohistochemical assays

#### Immunohistochemistry for Wnt-10b

**Figure 6:**
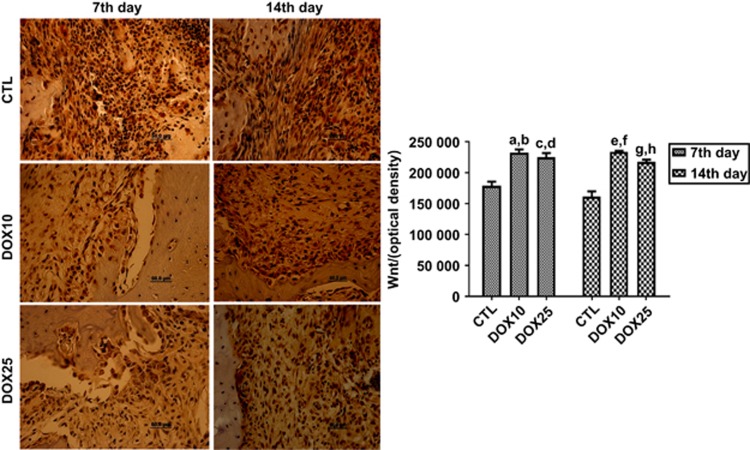
**Representative photomicrographs (× 400) of immunohistochemistry assays for Wnt-10b on the 7th and 14th days after tooth extraction, revealing significant increases in the number of immunopositive cells, as compared with that in the CTL in both protocols.** (**a**, **b**) *vs* CTL (7th and 14th days), *P*=0.000 1 and *P*=0.000 1, respectively; (**c**, **d**) *vs* CTL (7th and 14th days), *P*=0.000 5 and *P*=0.000 1, respectively; (**e**, **f**) *vs* CTL (7th and 14th days), *P*=0.000 2 and *P*=0.000 1, respectively; (**g**, **h**) *vs* CTL (7th and 14th days), *P*=0.005 6 and *P*=0.000 3, respectively (Two-way ANOVA and Tukey test for multiple comparisons). CTL, control; DOX10, doxycycline 10 mg·kg^−1^; DOX25, doxycycline 25 mg·kg^−1^.

Figure 6 shows representative photomicrographs of cells immunopositive for Wnt-10b on the 7th and 14th days after tooth extraction. Data quantification revealed significant and similar increases of 130% in the groups treated with DOX10 and DOX25 compared with the CTL group on the 7th day. A similar pattern (increases ranging from 140% to 150%) was observed in the DOX groups on the 14th day after surgery.

#### Immunohistochemistry for Dkk-1

**Figure 7:**
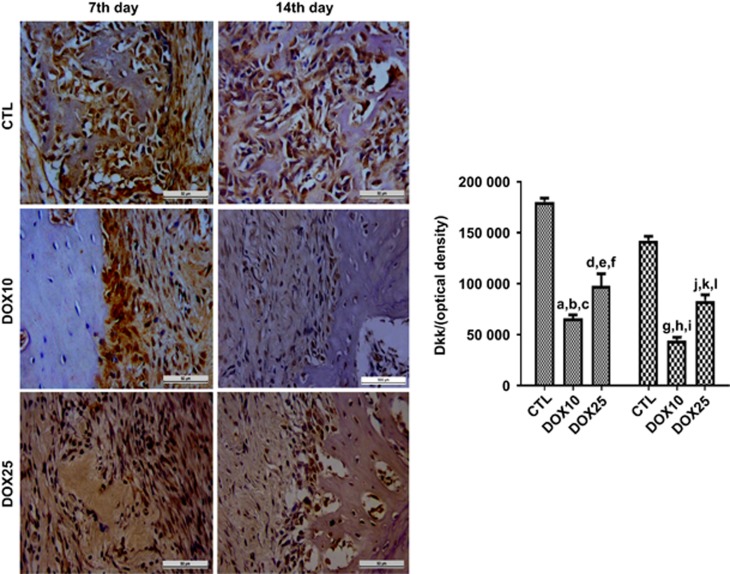
**Representative photomicrographs (× 400) of Dkk-1 immunopositive cells in the alveolar tissue on the 7th and 14th days after tooth extraction.** There were significant decreases in Dkk-1 immunostaining for both protocols after DOX treatment, as compared with that in the CTL. (**a**) *vs* CTL (14th day), *P*=0.007 4; (**b**–**d**) *vs* CTL (7th and 14th days) and *vs* DOX25 (7th day), *P*=0.000 1, *P*=0.000 1 and *P*=0.024 8, respectively; (**e**–**g**) *vs* CTL (7th and 14th days) and DOX10 (14th day), *P*=0.000 1, *P*=0.002 2 and *P*=0.000 4, respectively; (**h**–**j**) *vs* CTL (7th and 14th days) and *vs* DOX25 (14th day), *P*=0.000 1, *P*=0.000 1 and *P*=0.006 2, respectively; (**k**, **l**) *vs* CTL (7th and 14th days), *P*=0.000 1 and *P*=0.000 2, respectively (Two-way ANOVA and Tukey test for multiple comparisons). Dkk, Dickkopf; CTL, control; DOX10, doxycycline 10 mg·kg^−1^; DOX25, doxycycline 25 mg·kg^−1^.

Figure 7 shows representative photomicrographs of immunopositive cells for Dkk-1 on the 7th and 14th days after surgery. Quantification of data from the 7th day revealed significant decreases of 63% and 46% in the groups treated with 10 and 25 mg·kg^−1^ DOX, respectively, compared with the CTL group. Although this pattern was maintained on the 14th day, the number of immunopositive cells was 69% and 42%, for the DOX10 and DOX25 groups, respectively.

## Discussion

In the present work, DOX significantly decreased PMN and mononuclear inflammatory infiltrates in alveolus tissue on the 7th and 14th days after tooth extraction in the model of bone repair in rats. We have previously demonstrated that DOX presents potent anti-inflammatory and antioxidant effects.^20^ These properties are associated with inhibition of iNOS and TNF-alpha and are probably related to DOX’s actions on alveolar tissue inflammatory infiltrates, as shown in the present work.

At the lower dose, the DOX group, compared with the CTL group, exhibited significant increases in collagen fibres on the 7th and 14th days after tooth extraction, thus indicating better organisation and bone neoformation, as evaluated by PSR staining. Although no difference in the percentage of collagen fibres was observed between the DOX25 and CTL groups, the DOX25 group presented a significant number of type I collagen fibres. Type I collagen is primarily observed in bone, dentin, tendon, dermis and gingival tissues and is also the main extracellular component.^21, 22, 23^ Specific staining of the extracellular matrix components with PSR is especially helpful in studying tissue remodelling.^24^ The stain specifically binds to collagen fibrils of varying diameters, and was thus used to distinguish type I from type III collagen fibres.

The enhancement of birefringence promoted by the PSR-polarisation method is therefore specific for collagenous structures composed of aggregates of oriented molecules.^25^ A previous study ^26^ has demonstrated an increase in collagen fibre organisation over time. In addition, the authors have observed collagen maturation over 3, 7 and 14 days during orthodontic tooth movements in rats. DOX inhibits MMP enzymes and prevents bone loss.^27^ In addition, by increasing type I collagen fibres, DOX accelerates collagen maturation and bone repair, as observed in the present study.

MMPs are a family of enzymes that play a key role in maintaining and remodelling the extracellular matrix of connective tissue.^28^ Recently, low doses of orally administered DOX for 7 days have been found to enhance bone formation in the alveolar sockets of rats.^29^ The bone matrix is composed primarily of type I collagen fibres, which are a marker of osteoblast differentiation. Bone strength depends on more than quantity and mainly depends on quality, which is characterised by the geometry and shape of bones, the microarchitecture of trabecular bone, bone turnover, and the presence of minerals and collagen.^30^

We demonstrated that at both 10 and 25 mg·kg^−1^ doses, DOX significantly and dose-dependently decreased the number of osteoclasts, as compared with the CTL group on the 7th day post-surgery. A similar pattern was also observed on the 14th day. DOX induces osteoclasts apoptosis, which occurs independently of the inhibition of MMPs.^31^ Furthermore, DOX decreases mononuclear inflammatory infiltrates and osteoclast numbers, thereby preventing inflammatory bone resorption.^32^

Previously,^33^ fewer osteoclasts have been found to be present in DOX-treated animals in a surgically induced osteoclast recruitment model. Later evidence has indicated significant decreases in root resorption and the numbers of odontoclasts, osteoclasts and mononuclear cells on the root surface of DOX-treated rats.^34^ The authors concluded that DOX, at low doses, may have an inhibitory effect on orthodontically induced resorptive activity. Furthermore, DOX has been shown to effectively inhibit osteoclastogenesis and to affect mature osteoclast fate.^35^

In contrast, we demonstrated that DOX significantly and dose-dependently increased the number of osteoblasts, as compared with that in the CTL group, on the 7th day. Although an increase was also observed on the 14th day post-surgery, the effect was smaller, and no significant differences were observed among groups. Long-term exposure of human bone marrow osteoblastic cells to DOX induces a significant increase in the number of osteoblastic cells, thereby yielding a proportional amount of normal mineralised matrix.^36^ These effects suggest that this drug may potentially be used to increase bone formation. Other *in vitro* results have indicated the effect of DOX on osteoblastic proliferation and differentiation.^37^ The authors of that study have concluded that DOX appears to enhance maturation and differentiation, rather than proliferation, and may thus be beneficial in the treatment of periodontal disease and periodontal regeneration.

Wnt proteins play a very important role in several mammalian physiological processes, including embryogenesis, organ development and regeneration, and cell migration and proliferation.^38^ Osteogenesis is induced in response to bone morphogenic protein 2 (BMP2) stimulation and is sustained by Wnt signalling. However, the presence of Dkk-1, an inhibitor of Wnt signalling, results in osteogenesis inhibition, and during the repair process, the expression of many Wnt ligands and receptors is upregulated.^39, 40^ Wnt pathways regulate bone mass and are active during fracture repair, and an increase in their activity accelerates bone regeneration.^41^ Thus, the activation of Wnt pathways has the potential to improve bone healing, and its dysregulation greatly inhibits bone formation and healing processes.^17^

Evidence from preclinical studies has indicated that Dkk neutralization and/or Wnt signalling enhancement may prove effective in the treatment of bone pathologies.^42^ In addition, Wnt signalling by itself is a very attractive target for therapeutic interventions related to skeletal homoeostasis and bone repair.^43^ Thus, as we have shown here, Wnt signalling may be a potential target for DOX, and this result may stimulate translational studies towards including this drug in the clinic in the near future.

In the present work, we provided the first evidence that DOX inhibits the Dkk-1 pathway while activating Wnt signalling. These results were immunohistochemically evaluated through both the increase of Wnt-10b and the decrease in Dkk-1 in immunostained cells on the 7th and 14th days after tooth extraction. Although the exact mechanism of Wnt signalling during bone development is dependent on a complex microenvironment, data from several laboratories suggest that Wnt-3a, Wnt-5a, Wnt-7b and Wnt-10b are central to osteoblast differentiation.^44^

Clinical and experimental studies suggest a clear role for Wnt signalling in the regulation of bone formation, repair and remodelling. Thus, Wnt pathway activation accelerates bone regeneration.^45^ However, Dkk-1 counteracts Wnt-mediated effects on bone differentiation and adipogenesis. Thus, the neutralization of inhibitors of Wnt signalling, such as Dkk-1, is a promising therapeutic strategy in bone diseases.^46^

Wnt signalling is widely accepted to be required for the differentiation of osteoprogenitors into osteoblasts, and the Wnt pathway antagonist Dkk-1 causes bone destruction and the inhibition of bone repair.^47^ Recent evidence has indicated that selective inhibition of the Wnt pathway by Dkk-1 decreases osteoarthritis in mice.^48^ Furthermore, diseases such as periodontitis are characterised by inflammation and bone loss, and the activation of PMN cells leads to the release of proinflammatory cytokines and the recruitment of phagocytes and lymphocytes.^49^ Previously, we have demonstrated that DOX has potent anti-inflammatory activity.^20^ In addition, minocycline, the second generation tetracycline similar to DOX, offers protection in a model of periodontal disease in normal and diabetic rats(Ph.D. thesis, data not published).

## Conclusions

In the present study, we demonstrated that owing to its ability to increase osteoblast numbers and decrease osteoclast numbers, but mainly through its activation of Wnt-1b and neutralization of Dkk-1, DOX may be a potential candidate for use in bone repair in several pathologies, including periodontal diseases.

## Figures and Tables

**Table 1:** Histomorphometric analyses of the intensity of PMN inflammatory infiltrate in alveolar tissues at the 7th and 14th days after tooth extraction

	7th day			14th day		
Scores	CTL	DOX10	DOX25	CTL	DOX10	DOX25
Absence (0)	0	3	2	2	5	4
Discrete (1)	0	2	1	0	0	1
Moderate (2)	0	0	2	1	0	0
Intense (3)	5	0	0	2	0	0
Median	3 (3–3)	1(0–1)^a^	1(0–2)^b^	3 (3–3)	3 (2–3)	3 (3–3)

The results are presented as medians of scores from each group. a, b: as related to the CTL (Kruskal–Wallis and Dunn tests, *P*<0.05).

PMN, polymorphonuclear; CTL, control; DOX10, doxycycline 10 mg·kg^−1^; DOX25, doxycycline 25 mg·kg^−1^.

**Table 2:** Histomorphometric analyses of the intensity of mononuclear inflammatory infiltrate in alveolar tissues at the 7th and14th days after tooth extraction

	7th day			14th day		
Scores	CT	DOX10	DOX25	CT	DOX10	DOX25
Absence (0)	0	0	0	5	5	5
Discrete (1)	0	1	2	0	0	0
Moderate (2)	0	0	3	0	0	0
Intense (3)	5	4	0	0	0	0
Median	3 (3–3)	3 (1–3)	2 (1–2)	3 (3–3)	1 (0–1)	1 (0–1)

The results are presented as medians of scores from each group of five animals.

HE staining, × 400. CTL, control; DOX10, doxycycline 10 mg·kg^−1^; DOX25, doxycycline 25 mg·kg^−1^.

**Table 3:** Histomorphometric analyses of the degree of conjunctive tissue remodelling in alveoli at the 7th and 14th days after tooth extraction

	7th day			14th day		
Scores	CTL	DOX10	DOX25	CTL	DOX10	DOX25
Absence (0)	4	0	0	0	0	0
Discrete (1)	1	4	5	5	5	0
Moderate (2)	0	1	0	0	0	3
Intense (3)	0	0	0	0	0	2
Median	0(0–1)	1(1–2)^a^	1(1–1)^b^	1(1–1)	1(1–1)	2(2–3)

The results are presented as medians of scores in each group of five animals. a, b: as related to CTL.

HE staining, × 400. CTL, control; DOX10, doxycycline 10 mg·kg^−1^; DOX25, doxycycline 25 mg·kg^−1^.
